# Digital Quantification of Progesterone Receptor Expression in Meningiomas: Associations with WHO Grade and Clinicopathological Features

**DOI:** 10.5146/tjpath.2026.14663

**Published:** 2026-01-31

**Authors:** Yasemin Akca, Elif Büşra Gökçe

**Affiliations:** Department of Medical Pathology, Gaziantep University, Faculty of Medicine, Gaziantep, Türkiye

**Keywords:** Meningioma, Progesterone receptor, Digital pathology, H-score, Clinicopathological correlation

## Abstract

**Objective:** Progesterone receptor (PR) expression is a well-recognized marker in meningiomas, but manual immunohistochemical assessment is subjective and prone to variability. Digital pathology offers objective and reproducible quantification. Despite the availability of FDA-cleared digital PR scoring algorithms in some tumors like those of the breast, their implementation in meningiomas and systematic evaluation of their correlations with clinicopathological features remain scarce.

**Material and Methods:** We retrospectively analyzed 129 meningioma cases resected between 2018 and 2024. Digital PR expression was quantified using the FDA-cleared Ventana uPath PR 1E2 algorithm, originally developed for breast carcinoma. Subsequently, tumors were stratified by age, sex, WHO grade, histological subtype, and localization, while Ki-67 index and mitotic counts were also recorded. Associations with digital PR H-scores were evaluated using non-parametric tests, correlation analysis, and ordinal logistic regression.

**Results:** Digital PR H-scores progressively decreased across WHO grades, with Grade 1 tumors showing the highest values, followed by Grade 2 and Grade 3 (p=0.002). PR expression was inversely correlated with proliferative markers, including Ki-67 index (ρ = –0.42, p<0.001) and mitotic count (ρ = –0.35, p<0.01). No significant differences were observed by age or sex. Convexity meningiomas tended to have higher scores than skull base and spinal tumors.

**Conclusion:** Digital PR H-score assessment confirmed the inverse association between PR expression and WHO grade, as well as its correlation with proliferative activity. Using an FDA-cleared algorithm originally developed for breast carcinoma, this method provides objective and reproducible evaluation in meningiomas.

## INTRODUCTION

Meningiomas arise from arachnoid cap cells and are the most frequent primary intracranial tumors. They are graded by the WHO (Grade I–III) according to histopathological and, more recently, molecular criteria (1). Progesterone receptor (PR) expression is detected in a large proportion of meningiomas, generally ranging between 39% and 88%. Several studies have reported that PR levels tend to decline in parallel with increasing WHO grade, being highest in Grade I and lowest in Grade III tumors (2,3). Some studies have highlighted a strong inverse association between PR expression and proliferative activity in meningiomas, often measured by Ki-67 index or mitotic count (4). Specifically, PR negativity and elevated Ki-67 labeling index are more frequently observed in atypical and malignant variants, which supports their clinical value in risk stratification (5).

In most published reports, PR expression has been evaluated by manual IHC scoring, which is affected by inter-observer variation and the typical limitations of semi-quantitative assessment. Indeed, reviews note that IHC scoring of PR expression is often subjective and inconsistent (6), and studies evaluating inter-observer agreement in PR scoring report only moderate correlation (e.g. r = 0.63) between pathologists (7). Digital pathology provides an opportunity to assess PR expression more consistently using digital H-score methods, although its application in meningiomas has been relatively limited to date. Integrating digital PR scoring with clinicopathological parameters—including age, sex, tumor localization, molecular subtypes, and proliferation indices—may provide refined insights into tumor behavior and prognostication. This study therefore aims to evaluate the digital H-score of PR expression in a large series of meningiomas, and to elucidate its associations with WHO grade, histological subtype, location, Ki-67 index, mitotic activity, and demographic factors.

## MATERIAL AND METHODS

### Study Design and Cases

We retrospectively analyzed 129 meningioma cases resected at Gaziantep University between 2018 and 2024 to evaluate the associations between digital PR H-score and clinicopathological features. Ethics committee approval was obtained for this study (Approval No:2024/322).

### Digital PR Evaluation and H-score Calculation

Immunohistochemical staining for progesterone receptor (PR) was performed using the Ventana® PR (clone 1E2) assay. Whole-slide images of PR-stained sections were acquired with the Ventana DP200 digital slide scanner at ×20 magnification. Digital analysis was conducted with the FDA-cleared Ventana uPath PR 1E2 algorithm, originally developed for breast carcinoma. The algorithm has been previously compared with manual scoring to demonstrate its applicability to meningioma specimens (8). For each case, three representative regions of interest (ROIs) measuring approximately 5 mm² were selected from tumor areas by a pathologist, avoiding necrosis, hemorrhage, or non-neoplastic tissue. The software automatically quantified nuclear staining intensity as weak, moderate, or strong. The H-score was then calculated for each ROI using the formula (% weak × 1) + (% moderate × 2) + (% strong × 3), resulting in a continuous score ranging from 0 to 300. For each case, the mean of the three ROIs was used as the final digital PR H-score.

### Demographic Data

For statistical analysis, patients were stratified into three age groups: <40 years (n=24), 40–59 years (n=50), and ≥60 years (n=55). The study cohort included 90 female and 39 male patients (female-to-male ratio: ~2.3:1).

### Tumor Localization

Tumor localization was categorized into five groups: convexity (n=66; including frontal, parietal, temporal, occipital convexity and intraventricular meningiomas, as well as those described as arachnoid in origin), parasagittal/falx (n=9), skull base (n=36; including suprasellar, sphenoid wing, clinoidal, petroclival, olfactory groove, tentorial, foramen magnum, cerebellopontine angle, optic nerve–adjacent, and other cranial base sites including medulla oblongata), spinal (n=14; including cervical, thoracic, lumbar, and sacral as well as vertebra-related and posterior cerebral/cerebellar artery–adjacent lesions), and unknown/other (n=4).

### Histopathological Classification

Histopathological classification was performed according to the 2021 WHO Classification of Tumours of the Central Nervous System (CNS5) (1). Accordingly, meningiomas were assigned as Grade 1 (benign), Grade 2 (atypical/intermediate), or Grade 3 (anaplastic/malignant).

• Grade 1 tumors (n=97): meningothelial (n=27), transitional (n=50), fibrous (n=6), psammomatous (n=7), angiomatous (n=4), and microcystic (n=3).

• Grade 2 tumors (n=26): atypical meningiomas (n=23), chordoid meningiomas (n=2), and clear cell meningioma (n=1).

• Grade 3 tumors (n=6): anaplastic (n=5), one papillary meningioma (n=1). No rhabdoid subtype was identified.

### Proliferation Markers

Ki-67 proliferation index was recorded in all 129 cases (median 2%, range 1–40). Mitotic count was available for all tumors (median 1 per 10 HPF, range 0–47). Distribution according to WHO-relevant thresholds was as follows: 116 tumors with 0–3 mitoses, 10 tumors with 4–19 mitoses, and 3 tumors with ≥20 mitoses per 10 HPF.

The detailed distribution of demographic, clinical, and pathological characteristics of the study cohort is summarized in Table 1.

**Table 1:** Demographic and Clinicopathological Characteristics of Patients with Meningiomas (Compact Version)

**Variable**	**n**	**%**	**Details**
Age (years)	129	100	Mean 54.5 ± 15.3 (17–87)
<40	24	18.6	Median H-score 198
40–59	50	38.8	Median H-score 184
≥60	55	42.6	Median H-score 176
Sex			
Female	90	69.8	Median H-score 192
Male	39	30.2	Median H-score 188
Tumor localization			
Convexity	66	51.2	Highest scores
Skull base	36	27.9	
Spinal	14	10.9	Lowest scores
Parasagittal/falx	9	7.0	
Other/unknown	4	3.0	
WHO Grade I	97	75.2	Median H-score 206
Subtypes			Meningothelial 27; Transitional 50; Fibrous 6; Psammomatous 7; Angiomatous 4; Microcystic 3
WHO Grade II	26	20.2	Median H-score 157
Subtypes			Atypical 23; Chordoid 2; Clear cell 1
WHO Grade III	6	4.6	Median H-score 121
Subtypes			Anaplastic 5; Papillary 1
Proliferative markers			
Ki-67 index	129	100	Median 2% (1–40) ≤3%: n=86, 4–10%: n=21 >10%: n=22
Mitotic count	129	100	Median 1 (0–47) 0–3: 116; 4–19: 10; ≥20: 3

X**SD:** Standard deviation, **WHO: **World Health Organization, **HPF:** high-power field.

### Statistical Analysis

All clinicopathological variables, including age, sex, tumor localization, WHO grade, histological subtype, Ki-67 index, and mitotic count, were included in the statistical analyses. Continuous variables were summarized as median [IQR] and categorical variables as counts (%). Between-group comparisons of digital PR H-score across WHO grade, histological subtype, and location categories were performed using the Kruskal–Wallis test with Dunn’s post-hoc tests; p-values were adjusted for multiple comparisons using the Benjamini–Hochberg procedure. Correlations between H-score and proliferative markers (Ki-67, mitotic count) were assessed with Spearman’s rank correlation. Ordinal logistic regression models were fitted to examine the independent association between H-score and WHO grade (1<2<3) while adjusting for prespecified covariates. All tests were two-sided with α=0.05. Analyses were conducted in Python (pandas, SciPy, statsmodels).

## RESULTS

### Association of Digital PR H-score with WHO Grade

**Figure 1:**
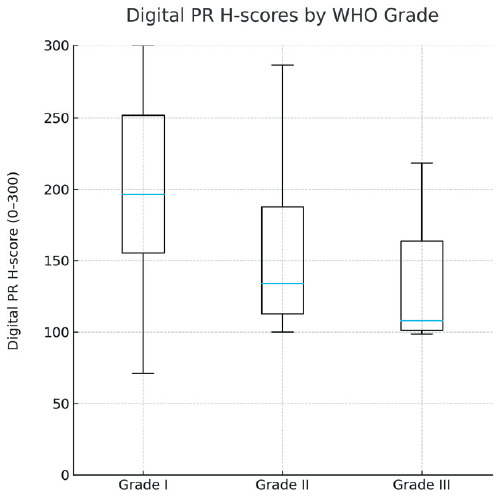
Boxplot of digital PR H-scores according to WHO grade. Digital PR H-scores (0–300) decrease with increasing grade (median: Grade I = 206, Grade II = 157, Grade III = 121; Kruskal–Wallis χ²=12.44, p=0.002; Dunn post-hoc: Grade I vs II and Grade I vs III, p<0.05). H-scores were computed using the FDA-cleared uPath PR 1E2 algorithm as the mean of three ~5 mm² ROIs per case.

Digital PR H-scores tended to decrease with higher WHO grade. The median H-score was 206 for Grade 1 tumors (n=97), 157 for Grade 2 tumors (n=26), and 121 for Grade 3 tumors (n=6). The overall difference among groups was statistically significant (Kruskal–Wallis χ²=12.44, p=0.002). Post-hoc pairwise testing demonstrated significant reductions in H-score between Grade 1 vs Grade 2 (p<0.05) and Grade 1 vs Grade 3 (p<0.05), whereas the difference between Grade 2 and Grade 3 was not statistically significant (p>0.05). These findings suggest that digital PR expression decreases as WHO grade increases, with the most notable difference seen between Grade 1 and the higher grades. The distribution of digital PR scores across the cohort is demonstrated in Figure 1.

### Association with Histological Subtypes

**Figure 2:**
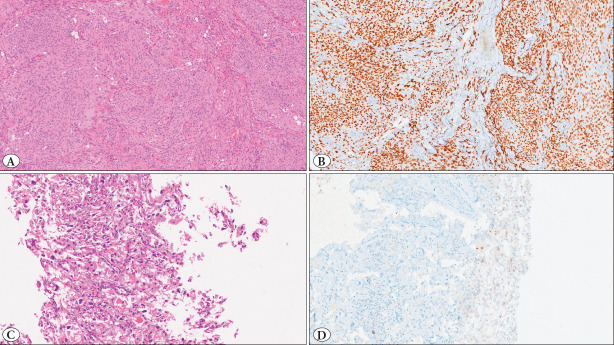
Representative histology and PR immunostaining in meningiomas. **A)** Grade I meningioma (meningothelial/transitional morphology), H&E, original objective ×20. **B)** Corresponding strong nuclear PR immunostaining (Ventana PR, clone 1E2), consistent with a high digital H-score. **C)** Grade I microcystic meningioma, H&E, original objective ×20. **D)** Corresponding weak/heterogeneous PR immunostaining, consistent with a low digital H-score.

When analyzed by histological subtypes according to the 2021 WHO classification, Grade 1 variants (meningothelial, transitional, fibrous, psammomatous, angiomatous, and microcystic) showed the highest digital PR H-scores. In contrast, Grade 2 tumors (atypical, chordoid, and clear cell) showed significantly lower scores, while Grade 3 tumors (anaplastic and papillary) had the lowest values. There was a statistically significant difference among the histological subtypes (Kruskal–Wallis p<0.01). Post-hoc analysis revealed that Grade 1 subtypes differed significantly from both Grade 2 and Grade 3 tumors (p<0.05 for each comparison), whereas the difference between Grade 2 and Grade 3 subtypes was not statistically significant. Within Grade I variants, fibrous and microcystic meningiomas exhibited comparatively lower digital PR H-scores (fibrous: median 114, IQR 105–148; microcystic: median 134, IQR 118–165) relative to other Grade I subtypes, although these differences did not reach statistical significance after multiple-testing correction. Representative examples are shown in Figure 2.

### Association with Tumor Localization

When stratified by anatomical site, digital PR H-scores varied across localization groups. The highest scores were observed in convexity meningiomas (n=66), followed by parasagittal/falx (n=9) and skull base tumors (n=36). Spinal meningiomas (n=14) and the miscellaneous/unknown group (n=4) had the lowest H-scores. The overall difference across groups reached statistical significance (Kruskal–Wallis p<0.05). However, post-hoc pairwise comparisons did not demonstrate statistically significant differences between individual localization groups after correction for multiple testing. These findings suggest that digital PR expression may be relatively higher in convexity meningiomas, whereas spinal and other rare sites show lower levels, although the small sample sizes limit definitive conclusions.

### Association with Age and Sex

When stratified by age categories, median H-scores were 198 in patients <40 years (n=24), 184 in those aged 40–59 years (n=50), and 176 in patients ≥60 years (n=55). Although there was a trend toward lower H-scores in older patients, the differences across age groups did not reach statistical significance (Kruskal–Wallis p>0.05).

Digital PR H-scores were similar between sexes, with median values of 192 in female patients (n=90) and 188 in male patients (n=39). No significant sex-related differences in H-scores were detected (Mann–Whitney p>0.05).

### Association with Proliferative Markers

Digital PR H-scores demonstrated a significant negative correlation with proliferative activity. H-score correlated inversely with Ki-67 index (Spearman’s ρ = –0.42, p<0.001), indicating that tumors with higher proliferative labeling tended to show lower PR expression. Similarly, an inverse correlation was observed with mitotic count (Spearman’s ρ = –0.35, p<0.01). When categorized, tumors with Ki-67 ≤3% exhibited higher H-scores compared to those with 4–10% or >10%, and a comparable trend was observed across increasing mitotic count strata (0–3, 4–19, ≥20 per 10 HPF), although groupwise differences were attenuated by the small number of high-mitotic tumors.

### Multivariable Analysis

In ordinal logistic regression including age, sex, localization, histological subtype, Ki-67 index, and mitotic count as covariates, digital PR H-score remained an independent predictor of WHO grade. Each 10-point increase in H-score was associated with a 15% reduction in the odds of being classified as a higher WHO grade (OR 0.85, 95% CI 0.76–0.95, p=0.004). Neither age nor sex showed significant associations with grade, whereas both Ki-67 index (p=0.01) and mitotic count (p=0.02) were independently linked to higher WHO grade. These findings indicate that lower digital PR expression is an independent marker of tumor aggressiveness.

All statistical comparisons of digital PR H-scores with clinicopathological variables, including WHO grade, histological subtype, tumor localization, age, sex, Ki-67 index, mitotic count, and multivariable regression results, are summarized in Table 2.

**Table 2:** Statistical Associations of Digital PR H-scores with Clinicopathological Variables

**Variable**	**Categories**	**Median H-score [IQR]**	**p-value / ρ**	**Interpretation**
WHO Grade	I (n=97)	206 [IQR]		
	II (n=26)	157 [IQR]		
	III (n=6)	121 [IQR]	0.002*	Significant decline with grade Post-hoc: I vs II, I vs III significant
Histological subtype	Grade I vs II vs III	-	<0.01*	Grade I > II & III Fibrous and microcystic lower (ns)
Tumor localization	Convexity, skull base, spinal, etc.	-	<0.05	Convexity highest; spinal lowest Pairwise ns after correction
Age group	<40, 40–59, ≥60	198, 184, 176	>0.05	Trend, not significant
Sex	Female vs Male	192 vs 188	>0.05	No difference
Ki-67 index	≤3%, 4–10%, >10%	-	<0.001*	Inverse correlation (ρ = –0.42)
Mitotic count	0–3, 4–19, ≥20	-	<0.01*	Inverse correlation (ρ = –0.35)
Multivariable analysis	Ordinal regression	-	OR 0.85 (95% CI 0.76–0.95), p=0.004	Independent predictor of grade Ki-67 p=0.01, Mitoses p=0.02

X**PR:** Progesterone receptor; **OR:** Odds ratio; **CI:** Confidence interval; **ρ:** Spearman correlation coefficient; **ns:** Not significant; **HPF:** High-power field. * Statistically significant.

A combined overview of demographic, pathological, and statistical findings is presented in Table 3, providing an integrated summary of the study results.

**Table 3:** Combined Overview of Demographics, Pathological Variables, and Key Statistical Findings

**Variable**	**Distribution**	**Key Findings**
Age	<40: 24 (18.6%) 40–59: 50 (38.8%) ≥60: 55 (42.6%)	Median H-scores 198 → 176 with age Decline trend, ns
Sex	Female: 90 (69.8%) Male: 39 (30.2%)	No significant difference (192 vs 188)
Localization	Convexity: 66 (51.2%) Skull base: 36 (27.9%) Spinal: 14 (10.9%) Falx: 9 (7.0%) Other: 4 (3.0%)	Convexity highest, spinal lowest Overall p<0.05, pairwise ns
WHO Grade	I: 97 (75.2%) II: 26 (20.2%) III: 6 (4.6%)	Median H-score: I=206, II=157, III=121 Significant decline (p=0.002)
Histological subtypes	Grade I: meningothelial 27, transitional 50, fibrous 6, psammomatous 7, angiomatous 4, microcystic 3 Grade II: atypical 23, chordoid 2, clear cell 1 Grade III: anaplastic 5, papillary 1	Grade I > II & III (p<0.01) Fibrous trend to lower
Ki-67 index	Median 2% (range 1–40) ≤3%, 4–10%, >10%	Inverse correlation (ρ = –0.42, p<0.001) Higher Ki-67 → lower PR
Mitotic count	Median 1/10 HPF (range 0–47) 0–3: 116 cases 4–19: 10 cases ≥20: 3 cases	Inverse correlation (ρ = –0.35, p<0.01) Higher mitoses → lower PR
Multivariable	Regression incl. age, sex, localization, subtype, Ki-67, mitoses	H-score independent predictor OR 0.85/10 points (95% CI 0.76–0.95, p=0.004) Ki-67 (p=0.01), Mitoses (p=0.02) also significant

X**PR:** Progesterone receptor; **ns:** Not significant; **OR:** Odds ratio; **CI:** Confidence interval; **ρ:** Spearman correlation coefficient; **HPF: **High-power field.

## DISCUSSION

In this study, we demonstrated that digital PR H-scores significantly decreased with increasing WHO grade in meningiomas. Grade 1 tumors exhibited the highest PR expression, whereas Grade 2 and particularly Grade 3 tumors showed markedly reduced scores. These findings confirm that loss of PR expression parallels tumor progression, consistent with previous studies employing manual scoring methods (3,9,10) although statistical significance was not reached in some studies (10). Importantly, our data extend these observations by demonstrating that a digital, algorithm-based evaluation yields robust and reproducible results.

The inverse correlation between PR expression and proliferative indices such as Ki-67 and mitotic count has been well documented. Our results corroborate these associations: tumors with higher Ki-67 or mitotic activity had significantly lower digital PR H-scores. This is in line with reports that PR-negative meningiomas are more likely to be atypical or anaplastic and to recur earlier (4,9).

Localization and histological subtype have been variably linked to PR expression in prior reports (2,3). In our series, convexity meningiomas tended to display higher PR scores compared with skull base and spinal tumors, although these differences did not remain significant in post-hoc tests, likely reflecting limited sample sizes. Among Grade 1 subtypes, fibrous meningiomas showed a trend toward lower PR expression, similar to earlier observations that fibrous morphology may be associated with lower PR levels (4). The microcystic Grade I variant likewise exhibited comparatively lower digital PR expression (median 134, IQR 118–165; vs overall Grade I median 206); however, these subtype differences did not persist after multiple-testing adjustment, likely owing to the rarity of the microcystic variant, and its small representation in our cohort (n=3). Collectively, these patterns point to subtype-specific heterogeneity in PR signaling.

A major strength of this study is the use of an FDA-cleared digital algorithm (Ventana uPath PR 1E2), which was originally validated for breast carcinoma. Applying this standardized platform to meningiomas reduces observer bias and increases reproducibility, providing a methodological advance over manual, semi-quantitative scoring. In addition, the adoption of an intensity and percentage-weighted H-score approach ensured further standardization and objectivity in quantifying PR expression. Furthermore, the relatively large sample size (n = 129) and the integration of multiple clinicopathological variables strengthen the robustness of our findings.

This study has some limitations. Molecular markers such as TERT promoter mutations or CDKN2A/B deletions, which are now included in the 2021 WHO classification, were not available for analysis (1). In addition, Ki-67 evaluation was performed manually rather than digitally, which may introduce variability. Finally, the small number of Grade 3 cases (n=6) limits the statistical power to detect subtle differences between atypical and anaplastic tumors.

Despite these limitations, our results confirm that declining PR expression is closely associated with higher WHO grade and tumor progression in meningiomas. Beyond methodological advantages, digital PR scoring thus holds promise as a clinically relevant biomarker that could aid in prognostication and, with further validation, inform therapeutic strategies targeting hormone-related pathways.

## CONCLUSION

This study confirms the inverse association between progesterone receptor expression and tumor grade in meningiomas and supports the feasibility of digital PR quantification as an objective method for this tumor type. Digital H-score assessment showed consistent performance and may serve as a useful adjunct to routine histopathological evaluation.

## Declaration of Generative AI Use

The authors declare that generative artificial intelligence (ChatGPT, OpenAI, San Francisco, CA, USA) was used to assist in language editing and improving the clarity of the manuscript. The authors reviewed, verified, and approved all content generated, and take full responsibility for the integrity and accuracy of the work.

## Funding

This research did not receive any specific grant from funding agencies in the public, commercial, or not-for-profit sectors.

## Conflict of Interest

The authors declare no conflicts of interest.
